# Validity and reliability of the Norwegian Adult Social Care Outcomes Toolkit (ASCOT CH4 and INT4) in three long-term care settings

**DOI:** 10.1186/s41687-026-00997-3

**Published:** 2026-01-27

**Authors:** Marijke Veenstra, Lisa Victoria Burrell, Ingeborg Strømseng Sjetne, Ann-Marie Towers, Maren Kristine Raknes Sogstad

**Affiliations:** 1 HSRU, Akershus University Hospital, Lillestrøm, Norway; 2 Centre for Care Research, Norwegian University of Science and Technology, Gjøvik, Norway; 3 Division for Health Services, Norwegian Institute of Public Health, Oslo, Norway; 4 Health and Social Care Workforce Research Unit, King’s College London, London, UK

**Keywords:** Quality of life, Quality ratings, Long-term care, Adult social care outcomes toolkit (ASCOT), Validity, Reliability

## Abstract

**Background:**

The Adult Social Care Outcomes Toolkit (ASCOT INT4 and CH4) for users of long-term care (LTC) services was translated into Norwegian. ASCOT assesses how LTC services may protect, improve or restore care recipients’ quality of life. The aim of this paper was to assess the psychometric properties of the Norwegian ASCOT for use in older adults in three LTC settings: nursing homes, sheltered housing and homecare.

**Methods:**

Cross-sectional data were collected between October 2022 and January 2024. The sample included 470 care recipients aged 65 years or older receiving LTC services in three municipalities in Norway. We used ASCOT INT4 and CH4 to collect information on eight domains of current and expected care related quality of life (CRQoL). We used exploratory factor analyses to assess the assumed one-dimensional structure of the ASCOT instruments and Cronbach’s alpha to describe internal consistency. Construct validity was examined using bivariate correlations of previously established associations among the different ASCOT domains and with health-related quality of life and sociodemographic factors. Differences between care settings were assessed with analyses of variance.

**Results:**

The analytical sample comprised of 184 residents from nursing homes,138 from sheltered housing and 148 persons receiving homecare services. Factor analyses supported a single underlying factor but factor loadings for the Dignity domain were sub-optimal (< 0.30) in nursing homes and sheltered housing. Cronbach’s alpha for current CRQoL was 0.70 in the total sample and ranged from moderate (0.67 in nursing homes) to good (0.71 in sheltered housing and 0.75 in homecare services). Bivariate correlations of ASCOT with relevant variables were largely consistent with previous studies and associations were higher for expected than current CRQoL. This assessment provides evidence to support the validity and reliability of the Norwegian ASCOT.

**Conclusions:**

Currently there are no other instruments assessing care-related aspects of older adults’ quality of life available in Norway. ASCOT provides a satisfactory reliable and valid measure of CRQoL in older adults receiving LTC, which goes beyond assessing functional capacity and health. Future research assessing different aspects of validity and reliability will further strengthen its applicability for evaluation purposes of Norwegian LTC.

## Background

In long-term care (LTC) among frail older persons, the main concern is often not health improvement but the need for support to compensate for the impact of impairments on quality of life (QoL) [1–3]. Conversely, the need for care services, the quality of the care received and the way services are evaluated as part of life, are essential features of QoL in this vulnerable population [4, 5]. The Adult Social Care Outcomes Toolkit (ASCOT) has been developed to address aspects of QoL most affected by formal LTC services [6]. The Toolkit is used to measure Care-Related Quality of Life (CRQoL) in a wide range of care settings, including care homes [7]. It can be applied in needs assessments and care planning [8, 9] and as a preference-based measure in economic evaluations [10, 11]. ASCOT emphasizes individual preferences and comprises both basic (e.g. food and drink, safety) and higher order domains (e.g. control and social participation). In doing so, it goes beyond a focus on health status, which is the core of most established instruments of health-related quality of life [12, 13]. The tool assesses how LTC services may protect, improve or restore care recipients’ QoL, where QoL is defined in terms of the ‘capability to achieve valuable functionings in life’ [14, 15]. As such, it focuses on ‘whether people are able to do what they would like to do’ [15], which goes beyond available Patient-Reported Experience Measures that directly inquire about care users’ reports on the care and the care process [16]. Originally developed for care settings in the UK [11], ASCOT has been translated for use in care settings in Denmark [17], Finland [2], Austria [18], The Netherlands [19] and Japan [20]. Accredited translations are available also in Spanish and Swedish. Recently, the face-to-face interview tool ASCOT-INT4 (Structured Interviews) and the four-level mixed-method tool ASCOT-CH4 (Care Homes) have been translated into Norwegian. With this new translation and adaptation, a potentially important measure for improving and benchmarking quality has become available for us.e in a range of Norwegian LTC settings.

The main aim of the current paper is to assess the reliability and construct validity of the Norwegian ASCOT for use in care recipients aged 65 years or older from three different LTC settings in Norway: nursing homes, homecare and sheltered housing. Earlier studies assessing the psychometric properties of the ASCOT instrument have indicated satisfactory internal reliability, test–retest stability and validity of the measure [9, 21, 22]. Cronbach’s alpha coefficients for the ASCOT-INT4 and ASCOT-CH4 were 0.70 and 0.77 respectively [2, 23] and test-retest reliability for all domains ranged from fair to substantial, with good test-retest reliability for a total index-score [24]. Validity refers to fundamental issues of how the construct of CRQoL should be related to observables, i.e., the eight ASCOT domains (see Table 1). These are the domains that are assumed to be most affected by care services and support. Each domain is captured by a single item with response levels that reflect four outcome states: ideal state, no unmet needs (but not to the desired level), some unmet needs and high unmet needs. The ideal state captures the concept of “capability” [14], whereas the other three states refer to levels of “functioning” [11]. Added together, the different domains constitute an overall CRQoL score. Previous validity assessments support a unidimensional structure of ASCOT’s CRQoL construct [11, 24], with some evidence for the Dignity domain as a potential second factor [2]. The Dignity domain is considered to differ from the other domains as it aims to reflect how the care process affects an individual’s sense of self-worth and self-respect, emphasizing the way care providers support and treat care recipients [11, 21]. It denotes the negative and positive psychological impact of support and care on the service user’s personal sense of significance.

Some ASCOT instruments also assess the *expected* CRQoL state, which refers to care recipients’ expectations on the domains in the absence of care services. Expected scores are an indicator of a person’s underlying needs for LTC. This way, the expected scores aim to reflect CRQoL in the counterfactual situation where the care recipient does not receive any care or support. Since a true measure of CRQoL without LTC services is impossible when services are already in place, this counterfactual approach is considered a pragmatic and valid way to obtain a type of baseline measure [25]. Expected scores can be subtracted from current CRQoL scores. These difference scores indicate CRQoL gain, which represents the estimated impact or benefit of the LTC services.

**Table 1:** Domains ASCOT

Domain	Definition
Food and drink	The service user feels he/she has a nutritious, varied and culturally appropriate diet with enough food and drink he/she enjoys at regular and timely intervals
Personal cleanliness and comfort	The service user feels he/she is personally clean and comfortable and looks presentable or, at best, is dressed and groomed in a way that reflects his/her personal preferences
Accommodation cleanliness and comfort	The service user feels their home environment, including all the rooms, is clean and comfortable
Personal safety	The service user feels safe and secure. This means being free from fear of break-ins, abuse, falling or other physical harm
Social participation and involvement	The service user is content with their social situation, where social situation is taken to mean the sustenance of meaningful relationships with friends, family and feeling involved or part of a community should this be important to the service user
Activity/Occupation	The service user is sufficiently occupied in a range of meaningful activities whether it be formal employment, unpaid work, caring for others or leisure activities
Control over daily life	The service user can choose what to do and when to do it, having control over his/her daily life and activities
Dignity	The negative and positive psychological impact of support and care on the service user’s personal sense of significance

Currently, no other instruments assessing care-related aspects of older adults’ QoL are available in Norway, and there is a need for measures that go beyond assessing functional capacity and health. Further validation of ASCOT, in a Norwegian LTC context, with a wide range of older users from different LTC services will provide valuable information on the psychometric properties of the instrument and inform Norwegian policy and practice about the availability of a valid tool to measure and monitor QoL in this vulnerable population.

## Norwegian care context

Of the LTC services investigated in the present study, home-based care services represent the lowest level of care, followed by sheltered housing, and with nursing homes representing the highest and most extensive level of care [26]. In Norway, 112,567 older people aged 67 + years received care services at home in 2024^1^, which constitutes the majority of LTC recipients (69 per cent). In addition, 22,197 older people (13 per cent of LTC recipients) live in sheltered housing facilities 2 and 28,947 (18 per cent of LTC recipients) live in an institution or a nursing home with personnel available 24 h a day [27]. Sheltered housing refers to assisted housing for persons with extensive LTC needs, but not so extensive that they require a permanent place in a nursing home. Sheltered housing usually refers to independent housing arrangements through rental apartments. They can include access to common areas, including social activities or meals, or specific adaptations to the home. People living in sheltered housing can apply for LTC services in their homes, which can range from limited support in daily activities to 24-hour services, depending on the individual’s needs. Municipalities are responsible for ensuring high quality public LTC provision to all their inhabitants. Most of the care is delivered by healthcare workers (including auxiliary nurses) and registered nurses. Service eligibility is needs-based, determined by municipal criteria in cooperation with the person’s primary physician. Homecare services supporting activities of daily living (self-care) are free of charge, whereas users are charged with co-payments, based on means-testing, to finance practical help in the home (e.g., help with cleaning, laundry, or cooking) as well as nursing home stays.

## Methods

### Sample

The study includes care recipients aged 65 years or older from nursing homes, sheltered housing and home-based care services. Exclusion criteria were receiving end-of-life care and receiving LTC for less than two weeks. Data were collected between October 2022 and January 2024. A representative from the research team contacted municipalities in the east of Norway with an invitation to participate in the study. Three urban municipalities with between 31,000 and 44,000 inhabitants agreed to participate. Subsequently, the head of each nursing home, sheltered housing and home-based service unit in these municipalities was contacted and received information about the study. Healthcare personnel at each unit were responsible for inviting care recipients, or ─ if care recipients were cognitively impaired and lacked capacity to consent ─ their next-of-kin, to participate in the study and sign the informed consent form. Recipients of home-based services had to be able to participate in a structured interview (INT4). This was not the case in sheltered housing or nursing homes, where also the mixed-methods mode of data collection (CH4) was available. Healthcare personnel from nursing homes and sheltered housing assessed whether a care recipient should be interviewed with the structured INT4 or the mixed-methods CH4 approach, and consequently whether next-of-kin should be contacted.

After excluding one person because of missing information on age, the sample in the current paper consisted of 470 care recipients aged between 65 and 100 + years, of whom 184 resided in nursing homes, 138 in sheltered housing and 148 received home-based care services. Healthcare personnel invited care recipients to participate in the study. 35% of the nursing home residents and 17% of the sheltered housing residents lacked capacity to consent. In these 87 instances, their proxies (next-of-kin) provided consent on their behalf. Interviews and observations were conducted by trained interviewers who were part of the research team.

### Measures

We used the Norwegian versions of the ASCOT INT4 and CH4 obtained by translation and back-translation. The INT4 toolkit [11] was used in face-to-face interviews with older adults receiving care services in their home. The CH4 measure was used for care recipients living in nursing homes (*N* = 64) or sheltered housing (*N* = 23) who had difficulty self-reporting. The CH4 toolkit collects data through structured observations and interviews, which form the basis for ratings of residents’ CRQoL [5, 23]. This approach enables the inclusion of all care recipients, regardless of their cognitive functioning. Data for the CH4 were collected by the trained interviewers through observations, conversations with residents, and proxy interviews with next-of-kin and staff. The interviewers triangulated this information and set the finale domain scores. Previous research [28] has shown that this approach is feasible and yields high levels of inter rater-reliability.

Table 1 provides an overview of the eight domains of the Norwegian ASCOT reflecting basic and higher order aspects of CRQoL [29]. Each domain is addressed by a single question with response options corresponding to four outcome states: ideal state [3], no unmet needs [2], some unmet needs [1] and high unmet needs (0) [11, 21]. In the absence of Norwegian preference weights, domain scores are weighted equally and added up to a total current CRQoL score (range: 0–24), with higher scores indicating a more positive rating of CRQoL. The ASCOT INT4 and CH4 tools used in this study also measure the expected CRQoL state for seven domains with response options corresponding to the same four outcomes. Expected CRQoL reflects care recipients’ expectations of what their CRQoL would be in each domain in the absence of care services. This counterfactual approach is not applied to the Dignity domain, which asks specifically about the process of care. In the absence of services, this domain is scored at the “no unmet needs” level (score 2) and included in the summed score for expected CRQoL [25]. Thus, the summed score for expected CRQoL can potentially range from 2 to 23.

In addition to the ASCOT measure, the questionnaire included a measure of health-related quality of life (HRQoL) and questions on socio-demographics. Educational attainment was grouped into a 3-level variable (Basic, Secondary and Higher education). For respondents with homecare services, information on living with a spouse or partner (yes/no) was registered. HRQoL was measured using the EQ-5D-3 L [30], which consists of five dimensions (mobility, self-care, usual activities, pain/discomfort, and anxiety/depression). The version used in the current study has three scoring levels, indicating ‘no problems’, ‘some problems’, and ‘extreme problems’. The instrument also contains a 20 cm vertical visual analogue scale, the EQ VAS, rating “your own health state today” on a 0 (worst imaginable) to 100 (best imaginable) scale. Using weight data from the UK, EQ-5D-3 L scoring profiles can be converted in value scores (utility scores), which reflect people’s preferences about how good or bad the state is. Scores of 1 indicate full health and 0 represents a state as bad as death. Negative scores indicate health states that are valued as worse than death.

### Statistical analyses

Internal consistency of the total CRQoL index was assessed with Cronbach’s alpha coefficient [31]. A coefficient of 0.70 or higher is generally considered acceptable reliability. Previous studies suggest that the ASCOT tools are best described as formative/mixed measurement models, with exploratory factor analysis (EFA) as the recommended approach to establish their structural characteristics, in particular for new translations of the ASCOT measure [32]. The current study thus uses EFA to assess aspects of construct validity of the current and expected ASCOT measures. In line with earlier findings, we anticipated a unidimensional structure with a single underlying factor of CRQoL [11, 23, 33]. We analysed the covariance matrix and used principal axis factoring. Bartlett’s test of sphericity and measures of Sampling Adequacy (MSA) were used to assess the appropriateness of EFA for the Norwegian ASCOT. Factors with eigenvalues > 1 and factor loadings > 0.30 were considered satisfactory [34]. We conducted EFAs for the total analytical sample including all three care settings and both interview forms, as well as separately for the ASCOT-INT4, comprising homecare recipients only. We also assessed the strength of the correlations among the different ASCOT domains. Spearman correlations between the individual items of the ASCOT measures exceeding 0.80 would suggest that the items have limited discriminatory validity.

*Convergent validity* was assessed by computing Spearman correlations of the overall and domain scores for EQ-5D-3 L and current and expected CRQoL. Spearman rank correlation coefficients are considered weak if they are below 0.30 and strong if they are above 0.50. In line with previous findings [2, 23], we anticipated weak to moderate positive correlations between the EQ-5D-3 L index and the CRQoL total scores. In addition, previous studies [23] have shown that the EQ-5D domains of Pain and Mobility are weakly correlated with the total CRQoL scores, whereas moderate correlations have been found for Anxiety & depression, Self-Care and Usual Activities [24]. Associations were found to be strongest for the expected CRQoL scores, i.e., the estimated QoL without care services. Moreover, several studies have shown the ASCOT domain of Personal safety to be positively associated with living with a spouse [13, 21]. In the current study, we expected to find similar associations as the ones specified above.

A skewed distribution of current CRQoL scores is anticipated when care services are effective. In that case, scores for current CRQoL should be clustered in the categories reflecting the ideal state or no unmet needs, with few people scoring in the states of some- and high-level of unmet needs [11]. This is especially expected for the domains measuring basic needs (e.g. Food & drink or Accommodation cleanliness) as care services are primarily targeting these areas. A marker of ASCOT’s discriminant validity is the ability of the ‘expected” CRQoL scores to distinguish according to care need [21], i.e., the greater the care need the lower a person’s ‘expected’ score. Thus, we would expect nursing home residents, who have greater care needs, to have significantly lower scores for expected CRQoL, followed by residents in sheltered housing and relatively higher scores for older persons receiving homecare services. We used one-way analyses of variance to test differences in current and expected CRQoL scores between the three care settings.

## Results

Sample characteristics are shown in Table 2. Average age in the total sample was 84.2 years and 69 per cent were women. 23% of the homecare recipients lived with their spouse/partner. Homecare recipients were significantly younger (p_ANOVA_<0.001) compared to residents in nursing homes and sheltered housing.

**Table 2:** Sample characteristics, total sample and across types of services

	Nursing home		Sheltered housing		Homecare		Total Sample	
	%/mean (SD)	*N*	%/mean (SD)	*N*	%/mean (SD)	*N*	%/mean (SD)	*N*
Age mean (SD)***	84.6 (7.9)	184	85.9 (8.9)	138	82.3 (7.5)	148	84.2 (8.2)	470
Sex								
Women	70.5	129	73.2	101	63.5	94	69.1	324
Men	29.5	54	26.8	37	36.5	54	30.9	145
Living with partner/spouse***								
Yes	0	0	6.8	9	23.0	34	9.3	43
No	100	184	93.2	123	77	114	90.7	421
Education								
Basic	49.1	79	49.3	66	36.4	51	45.1	196
Secondary	32.3	52	31.3	42	33.6	47	32.4	141
Higher	18.6	30	19.4	26	30	42	22.5	98
EQ5D-3L VAS mean (SD)*	60.1 (21.5)	174	63.3 (20.8)	129	56.7 (22.3)	141	59.9 (21.7)	444
EQ5D-3L utility mean (SD)***	0.50 (0.19)	175	0.63 (0.22)	134	0.59 (0.18)	143	0.57 (0.20)	452

*P < 0.05; **P < 0.01; ***P < 0.001

Table 3 presents the distribution of responses to the eight domains of current CRQoL. Responses distributed across all four states, although the distribution was skewed towards the more positive end of the response scale for all domains except for Activity. As anticipated [11], the basic needs domains of current CRQoL were particularly skewed. The distribution of scores for the domains of Control over daily life (P(χ^2^) = 0.043) and Activity (P(χ^2^) < 0.001) differed significantly across care settings.

**Table 3:** Distribution of responses to the domains of current CRQoL

Attribute	Nursing home	Sheltered housing	Home care	Total
	*N* (Percent)	*N* (Percent)	*N* (Percent)	*N* (Percent)
Control over daily life*				
I have as much control over my daily life as I want (score 3)	70 (38.3)	65 (47.1)	56 (37.8)	191 (40.8)
I have adequate control over my daily life (score 2)	61 (33.2)	50 (36.2)	46 (31.1)	157 (33.5)
I have some control over my daily life, but not enough (score 1)	42 (22.8)	21 (15.2)	42 (28.4)	105 (22.4)
I have no control over my daily life (score 0)	10 (5.4)	2 (1.4)	3 (2.0)	15 (3.2)
*Missing values*	1 (0.5)	0	1 (0.7)	2
*Personal cleanliness and comfort*				
I feel clean and am able to present myself the way I like (score 3)	129 (70.1)	99 (71.7)	108 (73.0)	336 (71.5)
I feel adequately clean and presentable (score 2)	52 (28.3)	35 (25.4)	32 (21.6)	119 (25.3)
I don’t feel adequately clean or presentable (score 1)	2 (1.1)	3 (2.2)	7 (4.7)	12 (2.6)
I don’t feel clean or presentable at all (score 0)	1 (0.5)	1 (0.7)	1 (0.7)	3 (0.6)
*Missing values*	0	0	0	0
*Food an drink*				
I get all the food and drink I like when I want (score 3)	121 (65.8)	97 (69.6)	120 (81.1)	337 (71.9)
I get adequate food and drink at OK times (score 2)	55 (29.9)	36 (26.1)	22 (14.9)	113 (24.1)
I don’ t always get adequate or timely food and drink (score 1)	7 (3.8)	6 (4.3)	4 (2.7)	17 (3.6)
I don’t always get adequate or timely food and drink, and I think there is a risk to my health (score 0)	1 (0.5)	0 (0)	1 (0.7)	2 (0.4)
*Missing values*	0	0	1 (0.7)	1
*Accommodation cleanliness and comfort*				
My home is as clean and comfortable as I want (score 3)	126 (68.5)	99 (71.7)	82 (55.4)	307 (65.5)
My home is adequately clean and comfortable (score 2)	49 (26.6)	35 (25.4)	52 (35.1)	136 (29.0)
My home is less than adequately clean and comfortable (score 1)	8 (4.3)	4 (2.9)	10 (6.8)	22 (4.7)
My home is not at all clean and comfortable (score 0)	1 (0.5)	0 (0)	3 (2.0)	4 (0.9)
*Missing values*	0	0	1 (0.7)	1
*Safety*				
I feel completely safe (score 3)	109 (59.2)	96 (69.6)	88 (59.5)	293 (62.3)
Generally, I feel safe, but not as safe as I’d like (score 2)	66 (35.9)	35 (25.4)	50 (33.8)	151 (32.1)
I feel less than adequately safe (score 1)	9 (4.9)	6 (4.3)	7 (4.7)	22 (4.7)
I don’t feel at all safe (score 0)	0 (0)	1 (0.7)	3 (2.0)	4 (0.9)
*Missing values*	0	0	0	0
*Social participation*				
I have as much social contact as I want with people I like (score 3)	54 (29.3)	61 (44.2)	60 (40.5)	175 (37.5)
I have adequate social contact with people (score 2)	62 (33.7)	34 (24.6)	43 (29.1)	139 (29.8)
I have some social contact with people, but not enough (score 1)	53 (28.8)	37 (26.8)	33 (22.3)	123 (26.3)
I have little social contact with people and feel socially isolated (score 0)	14 (7.6)	5 (3.6)	11 (7.4)	30 (6.4)
*Missing values*	1 (0.5)	1 (0.7)	1 (0.7)	2
Activity***				
I’m able to spend my time as I want, doing things I value or enjoy (score 3)	33 (17.9)	58 (42.0)	57 (38.5)	148 (31.7)
I’m able to do *enough* of the things I value or enjoy (score 2)	54 (29.3)	35 (25.4)	28 (18.9)	117 (25.1)
I do some of the things I value or enjoy but not enough (score 1)	78 (42.4)	37 (26.8)	51 (34.5)	166 (35.5)
I don’t do anything I value or enjoy (score 0)	18 (9.8)	8 (5.8)	10 (6.8)	36 (7.7)
*Missing values*	1 (0.5)	0	2 (1.4)	3
*Dignity*				
The way I’m helped and treated makes me feel better about myself (score 3)	113 (61.4)	81 (58.7)	90 (60.8)	284 (60.4)
The way I’m helped and treated does not affect the way feel about myself (score 2)	50 (27.2)	45 (32.6)	48 (32.4)	143 (30.4)
The way I’m helped and treated undermines the way I feel about myself (score 1)	12 (6.5)	8 (5.8)	7 (4.7)	27 (5.7)
The way I’m helped and treated completely undermines the way I feel about myself (score 0)	6 (3.3)	0 (0)	1 (0.7)	7 (1.5)
*Missing values*	3 (1.6)	4 (2.9)	2 (1.4)	9 (1.9)
Current CRQoL summated scores: Mean (SD)*	*18.4 (3.3)*	*19.6 (3.3)*	*18.9 (3.7)*	*18.9 (3.4)*
*Missing values*	4 (2.2)	4 (2.9)	3 (2.0)	11 (2.3)

*p < 0.05; ***p < 0.001

Appendix 1 presents the distribution of responses for expected CRQoL. Compared to current CRQoL, expected scores were more evenly distributed across the four states, reflecting that QoL would be poorer in the absence of care services. Still, among recipients of homecare services, more than half of the sample scored in the top two levels (no unmet needs) of the expected domain of Food & drink.

The proportion of missing values for each domain of current CRQoL was small: between 0 and 1.9 per cent (Table 3). This proportion was slightly higher for the domains of expected CRQoL, ranging from 2.1 to 4.7 per cent in the total sample (Appendix A). The proportion of missing values for domains of expected CRQoL was low among respondents receiving homecare services. All in all, 459 persons had valid responses on all eight domains of current CRQoL, and 434 persons had valid responses on the seven domains of expected CRQoL. Seven persons with residential care had missing values on all seven domains of expected CRQoL. The overall current CRQoL score had a negatively skewed distribution (Skewness − 0.72 and std. error = 0.11), suggesting a possible ceiling effect at the upper end of the scale. The overall expected CRQoL on the other hand had a positively skewed distribution (Skewness 0.64 and std. error = 0.12).

Results of the EFA are reported in Table 4. We first conducted EFA for the total sample including all eight domains of current CRQoL. Statistics for sampling adequacy (KMO = 0.79) and correlations between items (Bartlett’s test χ² [27] = 527.3, *p* < 0.001) were adequate. Measures of Sampling Adequacy (MSA) were all above 0.77. Principal Axis Factoring showed a clear single factor solution. Only the first factor had an eigenvalue > 1 and explained 38 per cent of the total variance. Seven domains had satisfactory factor loadings (> 0.30), but the Dignity domain had a factor loading of 0.22, which was below the threshold.

**Table 4:** Factor loadings and uniqueness (1-communalities) for current CrQoL (total sample and homecare services) and expected CRQoL (total sample) as measured by the one-factor solution for ASCOT

	Current CrQoL (*N* = 459)		Current CrQoL (without dignity) (*N* = 465)		Current CrQoL HomeCare (*N* = 145)		Expected CRQoL (*N* = 434)	
	Factor loadings	Uniqueness	Factor loadings	Uniqueness	Factor loadings	Uniqueness	Factor loadings	Uniqueness
Control over daily life	0.66	0.56	0.66	0.56	0.78	0.40	0.70	0.50
Social participation & involvement	0.65	0.58	0.65	0.58	0.58	0.66	0.62	0.62
Activity/Occupation	0.62	0.61	0.64	0.59	0.60	0.64	0.71	0.50
Personal safety	0.48	0.78	0.47	0.78	0.50	0.75	0.64	0.59
Personal cleanliness & comfort	0.42	0.82	0.42	0.82	0.47	0.78	0.74	0.45
Food & Drink	0.37	0.86	0.38	0.86	0.42	0.82	0.72	0.48
Accommodation cleanliness and comfort	0.33	0.89	0.33	0.89	0.43	0.82	0.60	0.64
Dignity	0.22	0.95			0.31	0.90		

We subsequently conducted a separate EFA for current CRQoL in respondents receiving homecare services (*N* = 145), all of whom had filled out ASCOT-INT4. These findings also supported the presence of a large first factor, explaining 32 per cent of the total variance. For this sample, all factor loadings, including the loadings for Dignity, were > 0.30, with most loadings being > 0.40. KMO was 0.79 and Bartlett’s test of sphericity was satisfactory χ ² [27] = 214.6, *p* < 0.001. All MSAs were above 0.73. Cronbach’s coefficient alpha for the eight-item scale of current CRQoL was 0.70 for the total sample and ranged from moderate (0.67 in nursing homes) to good (0.71 in sheltered housing and 0.75 in homecare services).

A third EFA was conducted for the total sample including the seven domains of expected CRQoL. All factor loadings were > 0.60 (Table 4). KMO was 0.88 and Bartlett’s test of sphericity was satisfactory χ ² = 1093.7, *p* < 0.001. All MSAs were above 0.85. Cronbach’s coefficient alpha for expected CRQoL in the total sample was 0.86 (0.77 in homecare services, 0.82 in nursing homes and 0.84 in sheltered housing).

Table 5 shows the bivariate Spearman correlations of current CRQoL and expected CRQoL scores with the EQ-5D-3 L summary scales and dimension scores, for the total sample and across the three LTC services contexts. All in all, bivariate correlations of CRQoL with the EQ-5D index and VAS scores were weak to moderate, as anticipated. However, among residents in sheltered housing, we found a strong correlation of the EQ-5D index score with current CRQoL (ρ = 0.53), which predominantly reflected the strong correlation with Anxiety & depression (ρ=-0.51). The EQ-5D dimensions of Pain and Mobility were weakly correlated with current CRQoL (ρ < 0.30). As anticipated, correlations of the EQ-5D index were consistently larger with expected than with current CRQoL. Strong correlations were found between expected CRQoL and the EQ-5D domains of Usual activities and Self-care. Conversely, correlations with Pain, Anxiety & depression and the VAS scores were higher for *current* CRQoL compared to those for *expected* CRQoL.

**Table 5:** Spearman rank correlations of current and expected CRQoL with EQ-5D-3 L domains and index. Total sample and stratified across healthcare services

	Nursing homes		Sheltered housing		Homecare services		Total sample	
	Current CRQoL (*N* = 168)	Expected CRQoL (*N* = 160)	Current CRQoL (*N* = 123)	Expected CRQoL (*N* = 114)	Current CRQoL (*N* = 136)	Expected CRQoL (*N* = 135)	Current CRQoL (*N* = 427)	Expected CRQoL (*N* = 409)
EQ-5D-3 L index	0.281**	0.443**	0.532**	0.495**	0.476**	0.412**	0.442**	0.469**
Mobility	-0.135*	-0.397**	-0.354**	-0.291**	-0.170*	-0.226**	-0.234**	-0.306**
Self-care	-0.135	-0.499**	-0.335**	-0.543**	-0.271**	-0.415**	-0.267**	-0.566**
Usual activities	-0.126	-0.533**	-0.345**	-0.417**	-0.357**	-0.623**	-0.289**	-0.595**
Pain	-0.100	-0.021	-0.305**	-0.152	-0.363**	-0.085	-0.239**	-0.028
Anxiety & depression	-0.351**	0.046	-0.508**	-0.389**	-0.279**	-0.068	-0.384**	-0.156*
EQ-5D-3 L VAS	0.245**	0.189	0.479**	0.435**	0.491**	0.440*	0.397**	0.245**
Living with spouse	na	na	na	na	-0.183*	-0.048		

na = not applicable; *p < 0.05 **p < 0.01

Table 5 also shows some noteworthy differences in correlations between the three care settings. For *current* CRQoL, correlations with the different EQ-5D-3 L domains were generally weak and lowest among nursing home residents, except for moderate correlations with Anxiety & depression (ρ=-0.35). Most corresponding correlations among residents in sheltered housing and homecare recipients were moderate. Moreover, in sheltered housing, bivariate correlations of the EQ-5D-3 L dimensions with *current* CRQoL were largely similar to corresponding correlations with *expected* CRQoL. In the other two care settings, correlations of the EQ-5D dimensions with current CRQoL differed markedly from those with expected CRQoL. For example, in nursing home residents, correlations of Usual Activities with current CRQoL were non-significant and weak (ρ=-0.13), but correlations with expected CRQoL were strong (ρ=-0.53). The ASCOT domain of Safety was assumed to be significantly and positively correlated with living with a spouse among recipients of homecare services. However, this correlation was weak and negative (ρ = -0.18).

Finally, we assumed that the need for LTC would be strongly and negatively associated with expected CRQoL [21]. All seven domain scores for expected CRQoL differed significantly across services setting, with recipients of homecare services overall showing the highest scores; i.e., lesser impact of LTC (see Appendix Table A). Mean expected CRQoL scores for nursing home residents were 5.5 (sd = 3.7), which was significantly lower (P_Anova_ < 0.001) compared to residents in sheltered housing (mean = 8.7 & sd = 5.3) and recipients of homecare services (mean = 11.9 & sd = 5.2) respectively.

## Discussion

The current study assessed aspects of reliability and validity of the Norwegian translation of the ASCOT instrument in a sample of older care recipients from nursing home, sheltered housing or homecare settings. The results indicated support for the unidimensional structure of the ASCOT measure of CRQoL and showed satisfactory psychometric qualities of the instrument for use in Norwegian LTC settings. Our findings suggested more favourable psychometric properties for current CRQoL in the sample of older persons receiving homecare services compared to persons in sheltered housing and nursing homes. One important explanation lies with the Dignity domain. In the two residential care settings, Dignity was, at best, weakly related to other domains of current CRQoL. A previous study using ASCOT-proxy also found low factor loadings for the Dignity domain [35]. Earlier research has shown that when rated by observers in residential care settings, the scoring of the Dignity domain may be prone to more subjective views than the other ASCOT domains [36]. In addition, in case of self-reports, it is considered a more cognitively demanding question to answer, asking residents to reflect on the impact of the way they are treated by staff. Internal consistency of the total scale for current CRQoL consisting of all eight domains was however deemed satisfactory, with Cronbach’s alpha ranging from moderate to good across the three LTC settings. Future studies addressing the structural characteristics of the ASCOT-CH4 in Norway should pay particular attention to the validity and reliability of the Dignity domain. Additional in-depth qualitative studies on how older Norwegian care recipients consider the impact of LTC on feelings of self-esteem and how they complete the Dignity item can provide important insights on its validity and on the need for adaptations. Expected CRQoL had good internal consistency in all three care settings and was strongly correlated with functional ability (the ability to perform usual activities and self-care). Construct validity of expected CRQoL was further supported by strong associations with LTC needs as reflected by the three LTC settings: CRQoL in the absence of care services was lowest for nursing home residents, followed by residents in sheltered housing and homecare recipients.

Our findings also suggested that ASCOT is feasible, given the small proportion of responses missing in each domain of current CRQoL, especially for the use of the INT4 in homecare settings. The proportion of missing values was higher for some higher-order domains of expected CRQoL in residential care settings. This suggests somewhat reduced feasibility of the counterfactual scoring methods in these settings. Distributions of current CRQoL scores in the basic needs domains were highly skewed, reflecting the predominant contribution of Norwegian LTC services to meet older adults’ basic needs. Similar skewness is also reported by studies from other countries, e.g., the UK [21].

We did not find that living with a spouse was associated with the ASCOT domain of Personal safety, referring to feelings of safety inside as well as outside the home. This lack of association was also reported in a previous study on the validity of the Dutch ASCOT [13]. This study suggested participants may have interpreted the item too narrowly, with a main focus on crime. In line with expectations based on previous findings from the UK [23] and Finland [2], our results showed weak to moderate correlations between the EQ-5D index scores and CRQoL, with the majority of correlations being stronger for expected than current CRQoL. These findings support the construct validity of the Norwegian ASCOT measure. For the EQ-5D dimensions of Self-care and Usual Activities, bivariate correlations with *expected* CRQoL were substantially higher than with *current* CRQoL, which may reflect the role of LTC in mitigating against the negative impact of poor functioning. Conversely, correlations of Anxiety/depression with *current* CRQoL were consistently higher than with *expected* CRQoL, with the largest discrepancy among nursing home residents. This may reflect a mental component of being in a situation where one becomes dependent upon LTC services. With a predominant focus on activities of daily living, mood and depression are often under-recognised and under-reported in older adults residing in care homes.

Strong correlations between current CRQoL and several domains of the EQ-5D were found in respondents living in sheltered housing and suggest a partial conceptual overlap of ASCOT with some of the health-dimensions measured by the EQ-5D. Despite this overlap, ASCOT captures additional information and can be used to complement the EQ-5D, also in the context of sheltered housing.

Limitations of this study are the lack of a nationally representative sample and lack of information on non-response. The key objective was to ensure that we included respondents from three different care settings in different municipalities. This way, our samples are sufficiently large to conduct analyses describing the ASCOT measure across LTC settings, but restricting sampling to three municipalities may have limited the external validity of the current findings. In all care settings, healthcare staff constituted the necessary link to invite older adults, or their proxies, to participate. This sampling procedure could introduce selection bias if healthcare staff would be prone to invite informants more positively inclined, and as such could challenge external validity of the findings. Data collection was time-consuming, especially in nursing home settings, lasting over a year, which reduces the feasibility of ASCOT. Ongoing work in the UK addresses this challenge of measuring CRQoL in care homes by piloting the use of the ASCOT-Proxy in digital care records [9]. A major advantage of the ASCOT-CH4 is that it avoids exclusion of persons who have difficulty with self-reporting by relying on proxy interviews with staff and family as well as on trained raters’ observations and conversations with residents. Despite high levels of inter-rater reliability, findings from previous research [23] have shown that care workers tend to over-estimate residents’ CRQoL, which could lead to information bias. By also relying on rater’s observations and conversations with residents, the CH-4 includes a way of accounting for residents’ experiences in the final CRQoL ratings. Moreover, we followed established recommendations to use EFA in assessing the structural characteristics of the newly translated ASCOT-INT4 version, which gives the advantages of comparability of the Norwegian version with other translations, and as such increases the relevance and informativeness of our paper. However, future research should consider the use of additional techniques to study the underlying relations between the ASCOT domains, e.g., network analysis [32], which does not rely on specifying the underlying measurement model. Finally, the current study assessed psychometric properties of the translated eight domains of the original ASCOT tool, which is developed for the social care sector in the UK. Future research should pay attention to the content validity of the ASCOT when using utility scores in the Norwegian LTC setting, where LTC to older adults predominantly involves the provision of healthcare services [27]. If key aspects of CRQoL constituting a person’s utility function are not represented by ASCOT, the instrument’s ability to provide an adequate valuation of care services may be sub-optimal for the Norwegian LTC setting [11]. Subsequent in-depth qualitative studies with end-users or an expert’s review could inform on the need for including additional domains to complement the original ASCOT measure in evaluating LTC services for older adults in Norway.

## Conclusion

The current study is the first to outline the psychometric properties of the Norwegian translation of the ASCOT toolkit for older adults in three different LTC contexts: nursing homes, sheltered housing and homecare services. Currently there are no other instruments assessing care-related aspects of older adults’ quality of life available in Norway, and ASCOT provides a satisfactory reliable and valid measure of CRQoL in older adults receiving LTC, which goes beyond assessing functional capacity and health. Future research assessing different aspects of validity and reliability will further strengthen its applicability for evaluation purposes of Norwegian LTC.

## Appendix A distribution of responses to the domains of expected CRQoL

**Table:** 

Attribute	Nursing home	Sheltered housing	Homecare	Total
	Frequency (Percent)	Frequency (Percent)	Frequency (Percent)	Frequency (Percent)
*Control over daily life*				
I would have as much control over my daily life as I want (score 3)	5 (2.7)	12 (8.7)	22 (14.9)	39 (8.3)
I would have adequate control over my daily life (score 2)	5 (2.7)	17 (12.3)	17 (11.5)	39 (8.3)
I would have some control over my daily life, but not enough (score 1)	38 (20.7)	40 (29.0)	51 (34.5)	129 (27.4)
I would have no control over my daily life (score 0)	125 (67.9)	61 (44.2)	56 (37.8)	242 (51.5)
*Missing values*	11 (6.0)	8 (5.8)	2 (1.4)	21 (4.5)
*Personal cleanliness and comfort*				
I would feel clean and am able to present myself the way I like (score 3)	6 (3.3)	38 (27.5)	46 (31.1)	90 (19.1)
I would feel adequately clean and presentable (score 2)	21 (11.4)	14 (10.1)	25 (16.9)	60 (12.8)
I would not feel adequately clean or presentable (score 1)	45 (24.5)	32 (23.2)	38 (25.7)	115 (24.5)
I would not feel clean or presentable at all (score 0)	105 (57.1)	51 (37.0)	39 (26.4)	195 (41.5)
*Missing values*	7 (3.8)	3 (2.2)	0	10 (2.1)
*Food an drink*				
I would get all the food and drink I like when I want (score 3)	5 (2.7)	20 (14.5)	74 (50.0)	99 (21.1)
I would get adequate food and drink at OK times (score 2)	16 (8.7)	23 (16.7)	22 (14.9)	61 (13.0)
I would not always get adequate or timely food and drink (score 1)	30 (16.3)	25 (18.1)	14 (9.5)	69 (14.7)
I would not always get adequate or timely food and drink, and I think there is a risk to my health (score 0)	127 (69.0)	64 (46.4)	36 (24.3)	227 (48.3)
*Missing values*	6 (3.3)	6 (4.3)	2 (1.4)	14 (3.0)
*Accommodation cleanliness and comfort*				
My home would be as clean and comfortable as I want (score 3)	5 (2.7)	15 (10.9)	29 (19.6)	49 (10.4)
My home would be adequately clean and comfortable (score 2)	12 (6.5)	18 (13.0)	29 (19.6)	59 (12.6)
My home would be less than adequately clean and comfortable (score 1)	49 (26.6)	35 (25.4)	36 (24.3)	120 (25.5)
My home would not at all be clean and comfortable (score 0)	110 (59.8)	65 (47.1)	52 (35.1)	227 (48.3)
*Missing values*	8 (4.3)	5 (3.6)	2 (1.4)	15 (3.2)
*Safety*				
I would feel completely safe (score 3)	7 (3.8)	21 (15.2)	21 (14.2)	37 (7.9)
Generally, I would feel safe, but not as safe as I’d like (score 2)	16 (8.7)	16 (11.6)	29 (19.6)	52 (11.1)
I would feel less than adequately safe (score 1)	57 (31.0)	41 (29.7)	46 (31.1)	133 (28.3)
I would not feel at all safe (score 0)	95 (51.6)	54 (39.1)	49 (33.1)	232 (49.4)
*Missing values*	9 (4.9)	5 (3.6)	3 (2.0)	16 (3.4)
*Social participation*				
I would have as much social contact as I want with people I like (score 3)	7 (3.8)	21 (15.2)	37 (25.0)	65 (13.8)
I would have adquate social contact with people (score 2)	16 (8.7)	16 (11.6)	30 (20.3)	62 (13.2)
I would have some social contact with people, but not enough (score 1)	57 (31.0)	41 (29.7)	41 (27.7)	139 (29.6)
I would have little social contact with people and feel socially isolated (score 0)	95 (51.6)	54 (39.1)	38 (25.7)	187 (39.8)
*Missing values*	9 (4.9)	6 (4.3)	2 (1.4)	17 (3.6)
*Activity*				
I would be able to spend my time as I want, doing things I value or enjoy (score 3)	5 (2.7)	21 (15.2)	40 (27.0)	66 (14.0)
I would be able to do *enough* of the things I value or enjoy (score 2)	17 (9.2)	19 (13.8)	28 (18.9)	64 (13.6)
I would do some of the things I value or enjoy but not enough (score 1)	64 (34.8)	50 (36.2)	51 (34.5)	165 (35.1)
I wouldn’t do anything I value or enjoy (score 0)	86 (46.7)	41 (29.7)	26 (17.6)	153 (32.6)
*Missing values*	12 (6.5)	7 (5.1)	3 (2.0)	22 (4.7)
Expected CRQoL summated scores; Mean (SD)^1^	5.5 (3.7)	8.7 (5.3)	11.9 (5.2)	8.5 (5.4)
*Missing values*	16 (8.7)	15 (10.9)	5 (3.4)	36 (7.7)

^1^Expected Dignity scores are set to “2” for all respondents; ***p < 0.001

## Data Availability

Restrictions apply to the availability of these data, which were used under license for the current study, and are therefore not publicly available but stored on a secure server (TSD) with access restricted to the researchers involved in the research project (grant number 300654). Data are available from the authors upon reasonable request to the principal investigator (MS) and with permission of the Regional Committee for Medical and Health Research Ethics in Norway.

## Footnotes

1 Including practical help and healthcare in the home (e.g., help with selfcare including bathing, dressing etc.)

2 Dwellings for nursing and care purposes.

## Abbreviations

ASCOT (INT4): Adult Social Care Outcomes Toolkit ─ four level interview schedule
ASCOT (CH4): Adult Social Care Outcomes Toolkit ─ Care Homes, 4 outcomes states
LTC: Long term care
QoL: Quality of Life
CRQoL: Care Related Quality of Life
EQ5D-3L: The EuroQol five-dimensional three-level questionnaire
EFA: Exploratory Factor Analysis
MSA: Measure of Sampling Adequacy
